# Anxiety and academic burnout in migrant children: a chain mediation model of positive psychology and self-concept

**DOI:** 10.3389/fpsyg.2026.1768621

**Published:** 2026-02-19

**Authors:** Xiang Ji, Tao Wei, Yu Zuo, Miaomiao Wang

**Affiliations:** 1 College of Teacher Education, Weifang University, Weifang, China; 2 Weifang Gaoxin Jinma School, Weifang, China; 3 Xinjiang Key Laboratory of Mental Development and Learning Science, Urumqi, China

**Keywords:** academic burnout, anxiety, migrant children, positive psychology, self-concept

## Abstract

**Background:**

This study examined the impact of anxiety on academic burnout among migrant children who changed schools during the middle-to-upper primary grades. It further investigated the independent and sequential mediating roles of positive psychological capital and self-concept within this relationship.

**Methods:**

A cross-sectional survey was conducted with 473 migrant children in grades 5 and 6. The Spence Children's Anxiety Scale (SCAS), Positive Psychological Capital Questionnaire (PPQ), Piers-Harris Children's Self-Concept Scale (PHCSS), and Adolescent Learning Burnout Inventory (ALBI) were administered. Data analysis included common method bias assessment, descriptive and correlational analyses, hierarchical regression, and bootstrap mediation analysis.

**Results:**

Anxiety showed a significant positive association with academic burnout. Both positive psychological capital and self-concept independently mediated this relationship. Furthermore, a significant chain-mediating effect was observed: anxiety was linked to lower positive psychological capital, which was associated with a weaker self-concept, ultimately contributing to higher levels of academic burnout.

**Conclusion:**

The findings suggest that internal protective resources play crucial roles in the link between anxiety and academic burnout among migrant children. Interventions aimed at reducing academic burnout may benefit from a dual focus on mitigating anxiety while proactively fostering positive psychological capital and a healthy self-concept.

## Introduction

Migrant children in China, defined as school-age children who relocate with their parents to cities without local household registration (Ministry of Education of the People's Republic of China, 2012), face unique developmental challenges. This study focused on a specific subgroup: children who transferred from rural to urban schools during middle-to-upper primary grades. While this transition often involves improved parental care, it also presents significant difficulties in curriculum alignment, social integration, and psychological adaptation, predisposing these children to various psychological issues (Wei, 2018). Such problems can lead to declining academic engagement and performance, potentially fostering sustained alienation from mainstream education (Sirin, 2005).

Existing research on migrant children has extensively documented generalized psychological problems linked to environmental hardships. However, critical gaps remain. First, there is limited mechanistic investigation into how broader psychological distress, such as anxiety, becomes specifically internalized as academic burnout—a core form of academic maladjustment. Second, the literature predominantly adopts a deficit-oriented perspective, overlooking the potential protective functions of children's internal psychological resources. Specifically, the roles of positive psychological capital as an active resilience factor and self-concept as a core cognitive framework of self-worth remain underexplored through empirical models. Consequently, shifting the research focus from problem description to mechanism exploration, and from a deficit to a strength-based perspective, is imperative. Systematically analyzing the formation mechanism of academic burnout and exploring protective pathways have thus emerged as urgent scholarly priorities.

Academic burnout, characterized by exhaustion, cynicism, and reduced efficacy due to academic pressures (Xu and Yu, 2010), a process consistent with theoretical models linking academic stressors to maladaptive outcomes (Pekrun et al., 2009). Furthermore, perceived discrimination has been shown to significantly undermine the mental health of this population (Fan and Lu, 2020). Positive psychology emphasizes leveraging individual strengths to cultivate positive psychological resources (Diener, 2000), which may buffer against burnout. Concurrently, a clear and positive self-concept is directly associated with better academic adjustment and lower burnout (Li et al., 2021). For migrant children with experiences of inadequate care or adjustment difficulties, these internal resources may be particularly salient (Cheng and Sun, 2015). This study therefore investigated the relationships among anxiety, academic burnout, positive psychological capital, and self-concept in mid-primary school migrant children, providing a vital entry point for improving their mental health and reducing burnout.

Anxiety in migrant children, as conceptualized in major diagnostic frameworks (American Psychiatric Association, 2013), constitutes a chronic stress response triggered by identity conflict, social exclusion, and future uncertainty during the rural-urban transition (Liu, 2017). Research confirms that anxiety consumes cognitive resources, impairs learning, and fosters avoidant coping, leading to higher academic burnout in this population (Zhang et al., 2010; Xu et al., 2022). Therefore, it was hypothesized (*H*1) that anxiety would be positively associated with academic burnout.

Positive psychological capital—comprising self-efficacy, resilience, optimism, and hope (Luthans et al., 2007)—serves as an internal buffer system. Studies indicate that higher levels of positive psychological capital are linked to lower academic burnout, as these resources help individuals cope with stress and maintain engagement (Zhang et al., 2018; Piao, 2019). Thus, it was hypothesized (*H*2) that positive psychological capital would mediate the relationship between anxiety and academic burnout.

Self-concept, the multifaceted cognitive and evaluative understanding of oneself, regulates psychological activities and behavior. Unfavorable self-concept is associated with learning and social difficulties (Tang, 1999), while positive self-concept dimensions correlate negatively with academic burnout (Zhao, 2022; Pan, 2024). This suggests that self-concept may also serve as a mediator. Consequently, it was hypothesized (*H*3) that self-concept would mediate the relationship between anxiety and academic burnout.

Furthermore, theoretical frameworks and emerging research suggest that these protective resources may function sequentially. Positive psychological capital may influence burnout partly by enhancing self-concept (Liu, 2021; Han et al., 2022). Therefore, it was hypothesized (*H*4) that positive psychological capital and self-concept would play a chain-mediating role in the relationship between anxiety and academic burnout.

In summary, this study aimed to examine the impact of anxiety on academic burnout among migrant children and to test the independent and chain-mediating roles of positive psychological capital and self-concept, thereby providing a theoretical and empirical foundation for strength-based interventions.

## Methods

### Overall research methodology

A cross-sectional survey design was employed to investigate the relationships among the studied variables in mid-primary school migrant children. Data were collected via a one-time administration of standardized self-report questionnaires.

### Participants

Participants were recruited from fifth and sixth grades in an elementary school in Weifang City using cluster sampling. To avoid stigmatization, no distinction was made between migrant and non-migrant students during survey administration. Migrant children were identified *post-hoc* based on a school-provided list. Inclusion criteria were: (1) being a migrant child who had transferred during fifth or sixth grade, and (2) having a signed parental informed consent form. Questionnaires were screened for validity, excluding those with unreasonably short completion times, patterned responses, random answering, or missing items. From 490 initially collected questionnaires, 473 valid responses were retained (effective rate: 96.5%). The final sample included 244 boys (51.6%) and 229 girls (48.4%); 261 were in fifth grade (55.2%) and 212 in sixth grade (44.8%).

### Measures

#### Anxiety

The Chinese version of the Spence Children's Anxiety Scale (SCAS; Spence, 1999) was used. This 38-item scale measures six dimensions of anxiety on a 4-point scale. Higher total scores indicate higher anxiety levels. In this study, Cronbach's α was 0.91.

#### Positive psychological capital

The Chinese version of the Positive Psychological Capital Questionnaire (PPQ; Zhang et al., 2010) was administered. This 26-item scale assesses self-efficacy, resilience, hope, and optimism on a 5-point Likert scale. Higher scores indicate higher positive psychological capital. Cronbach's α was 0.93.

#### Self-concept

The Chinese version of the Piers-Harris Children's Self-Concept Scale (PHCSS; Piers and Harris, 1969) was used. This 80-item scale measures six dimensions on a 5-point scale. Higher scores indicate a more positive self-concept. Cronbach's α was 0.89.

#### Academic burnout

The Chinese version of the Adolescent Learning Burnout Inventory (ALBI; Wu and Dai, 2007) was employed. Although named a “Learning Burnout” inventory, the ALBI measures the core dimensions of academic burnout (exhaustion, cynicism, reduced efficacy) and has been validated for use with Chinese school-aged children, including primary school students. For conceptual consistency, the measured construct is referred to as academic burnout throughout this paper. This 16-item scale uses a 5-point Likert scale, with higher scores indicating more severe burnout. Cronbach's α was 0.93.

### Procedure

Prior to data collection, informed consent was obtained from teachers and parents/guardians. The study's purpose was explained to the children in an age-appropriate manner. They were informed that participation was voluntary, anonymous, and confidential, and that they could withdraw at any time. Verbal assent was obtained from each child before questionnaire distribution. Questionnaires were administered in paper-and-pencil format during regular school hours under teacher supervision. Completion time ranged from 5 to 15 min, and all questionnaires were collected on-site.

### Data analysis

Preliminary analyses included Harman's single-factor test to assess common method bias and computation of descriptive statistics and Pearson correlations. The main analyses consisted of two parts. First, hierarchical regression was conducted to examine direct effects, controlling for gender and grade. Second, a serial mediation model (Anxiety → Positive Psychological Capital → Self-Concept → Academic Burnout) was tested using the PROCESS macro for SPSS (Model 6; Hayes, 2018). Bias-corrected bootstrapping with 5,000 resamples was employed to generate 95% confidence intervals for all indirect effects.

## Results

### Common method bias test

Harman's single-factor test was conducted on all measurement items. An unrotated exploratory factor analysis extracted 24 factors with eigenvalues greater than 1. The variance explained by the first factor was 27.15%, below the critical threshold of 40%, indicating that significant common method bias was not a concern.

### Descriptive statistics and correlation analysis

Means, standard deviations, and correlations for all variables are presented in Table 1. Anxiety was significantly negatively correlated with positive psychological capital and self-concept, and significantly positively correlated with academic burnout. Positive psychological capital was significantly positively correlated with self-concept and significantly negatively correlated with academic burnout. Self-concept was significantly negatively correlated with academic burnout. Gender was positively correlated with anxiety and negatively correlated with positive psychological capital. Grade level was positively correlated with anxiety and academic burnout, and negatively correlated with positive psychological capital and self-concept. Therefore, gender and grade were controlled in subsequent analyses.

**Table 1:** Descriptive statistics and correlation analysis results (*N* = 473).

**Variable**	***M***	***SD***	**1**	**2**	**3**	**4**	**5**
1 Gender	1.48	0.50					
2 Grade	1.46	0.52	−0.01				
3 Anxiety	2.74	0.72	0.11^*^	0.23^**^			
4 Pos. Psych. Cap.	4.03	0.74	−0.08^*^	−0.22^**^	−0.58^**^		
5 Self-concept	4.00	0.62	−0.01	−0.22^**^	−0.62^**^	0.81^**^	
6 Learning burnout	2.11	0.67	−0.01	0.19^**^	0.46^**^	−0.65^**^	−0.65^**^

^*^p < 0.05, ^**^p < 0.01. The same notation applies hereafter. Pos. Psych. Cap., positive psychological capital.

### Gender difference test

**Figure 1:**
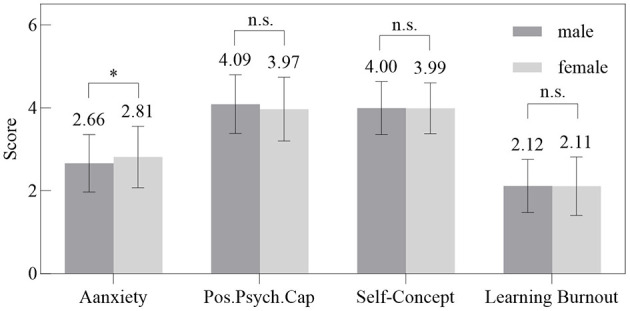
Gender differences in anxiety, positive psychological capital, self-concept, and learning burnout. *p < 0.05, **p < 0.01, ***p < 0.001. Scores are presented as mean values. Anxiety was measured on a 4-point scale; positive psychological capital, self-concept, and learning burnout were measured on 5-point scales.

Independent samples *t*-tests revealed that girls scored significantly higher than boys on anxiety (*t* = −2.28, *p* < 0.05). No significant gender differences were found for positive psychological capital (*t* = 1.81, *p* > 0.05), self-concept (*t* = 0.12, *p* > 0.05), or academic burnout (*t* = 0.14, *p* > 0.05). These results are summarized in Figure 1.

### Tests of main effects and chain-mediating effects

**Figure 2:**
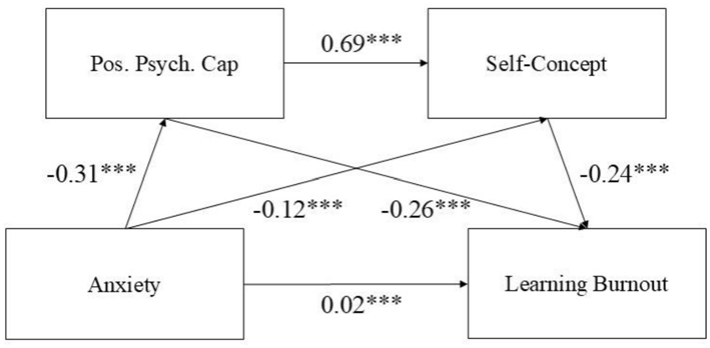
Chain-mediating effect model of positive psychological capital and self-concept. ***Indicates *p* < 0.001.

Hierarchical regression analysis, controlling for gender and grade, showed that anxiety significantly positively predicted academic burnout (β = 0.02, *p* < 0.001). Anxiety also showed significant negative direct effects on both positive psychological capital (β = −0.31, *p* < 0.001) and self-concept (β = −0.12, *p* < 0.001). Both positive psychological capital (β = −0.26, *p* < 0.001) and self-concept (β = −0.24, *p* < 0.001) significantly negatively predicted academic burnout. Positive psychological capital significantly positively predicted self-concept (β = 0.69, *p* < 0.001). The full model with standardized path coefficients is presented in Figure 2.

The bootstrap mediation analysis results are presented in Table 2. The total effect of anxiety on academic burnout was significant (effect = 0.18, 95% CI [0.15, 0.21]). The direct effect was not significant (effect = 0.02, 95% CI [−0.02, 0.05]). The total indirect effect was significant (effect = 0.16, 95% CI [0.13, 0.20]). Specifically, the indirect paths through positive psychological capital alone (effect = 0.08, 95% CI [0.05, 0.12]) and through self-concept alone (effect = 0.03, 95% CI [0.01, 0.05]) were both significant. Furthermore, the chain-mediating path through positive psychological capital then self-concept was also significant (effect = 0.05, 95% CI [0.03, 0.08]).

**Table 2:** Tests of mediating effects.

**Effect**	**Effect size**	**Boot*SE***	95% CI	
			**LLCI**	**ULCI**
Total effect	0.18	0.02	0.15	0.21
Direct effect	0.02	0.02	−0.02	0.05
Total indirect effect	0.16	0.02	0.13	0.20
Anxiety → Pos. Psych. Cap. → learning burnout	0.08	0.02	0.05	0.12
Anxiety → self-concept → learning burnout	0.03	0.01	0.01	0.05
Anxiety → Pos. Psych. Cap. → self-concept → learning burnout	0.05	0.01	0.03	0.08

Pos. Psych. Capital, positive psychological capital. Confidence intervals were bias-corrected based on 5,000 bootstrap samples.

## Discussion

The present findings illuminate the complex psychosocial processes linking migration-related anxiety to academic burnout among Chinese migrant children in the middle-to-upper primary grades. The confirmed positive association between anxiety and academic burnout (*H*1) aligns with and extends previous research documenting the detrimental effects of chronic stress on academic engagement in vulnerable populations (e.g., Liu et al., 2015; Zhang et al., 2021). While prior studies have established that migrant children experience higher levels of both anxiety and burnout compared to their non-migrant peers, this study specifies the nature of this relationship within a mechanistic model. Our results suggest that anxiety is not merely a comorbid condition but may function as a key antecedent that initiates a cascade of internal resource depletion, ultimately manifesting as the emotional exhaustion, cynicism, and reduced efficacy characteristic of burnout. This finding advances the literature by moving beyond descriptive correlation to position anxiety as a critical upstream factor in the etiology of academic maladjustment for this specific group, thereby identifying a precise target for preventive intervention.

The mediation through positive psychological capital (*H*2) underscores the vital buffering role of internal strengths, a perspective often overshadowed by the field's predominant focus on risk factors (Han et al., 2022). a finding corroborated by recent research on migrant children (Chen et al., 2023). Consistent with the conservation of resources theory (Hobfoll et al., 2018), anxiety appears to deplete key psychological resources such as hope, self-efficacy, resilience, and optimism. This erosion directly impairs a child's capacity for goal-directed thinking and adaptive coping, mechanisms previously implicated in burnout (Jiang and Li, 2018; Honicke and Broadbent, 2016). However, prior research on migrant children has frequently treated positive psychological capital as a static correlate or outcome. Our analysis contributes a more dynamic understanding by empirically demonstrating its role as a *mediating pathway* through which anxiety exerts its influence. This novel insight refines theoretical models by specifying that the protective function of positive capital lies not only in its direct effect but also in its susceptibility to being compromised by anxiety, thereby explaining variance in burnout susceptibility under similar stress levels.

Similarly, the mediating role of self-concept (*H*3) reveals a critical cognitive-affective pathway. Anxiety, stemming from identity conflicts and adaptation pressures (Liu, 2017), can disrupt and distort the developing self-system. This aligns with models suggesting that negative self-evaluations and poor interoceptive awareness fuel emotional exhaustion (Paulus and Stein, 2010; Li et al., 2012). While studies have linked poor self-concept to burnout in adolescents (Zhao, 2022; Pan, 2024), few have explicitly tested it as a mediator in the context of migration-related anxiety. Our finding that self-concept mediates this relationship provides empirical support for the theoretical proposition that anxiety internalizes as negative self-appraisals, which in turn catalyze disengagement and burnout. This positions self-concept not merely as a personal characteristic but as a pivotal internal processing mechanism that translates external stressors into academic disaffection.

The most significant contribution of this study is the identification of a sequential chain-mediation effect (*H*4). The model posits that anxiety first undermines positive psychological capital, which subsequently impedes the formation of a healthy self-concept, culminating in heightened academic burnout. This finding synthesizes and extends two previously somewhat separate strands of research: one on resource conservation (e.g., Hobfoll et al., 2018) and another on cognitive self-structures (e.g., Judge et al., 1997). It proposes a specific order of operations: the depletion of agentic, future-oriented resources (hope, efficacy) precedes and contributes to the deterioration of the core cognitive-evaluative self-system. This chain model offers a more nuanced and integrated theoretical framework than independent mediation, explaining *how* the erosion of positive states can destabilize the self-concept. It suggests that interventions solely targeting negative self-views may be insufficient if the foundational reservoirs of psychological capital are not simultaneously replenished.

The gender difference observed—where girls reported higher anxiety but showed no difference in other constructs or burnout—adds further nuance. This pattern supports the notion that gender may modulate the initial emotional reactivity to migration stress, potentially due to socialization differences in emotional expression (Li and Chen, 2013; Xiao et al., 2020). Crucially, however, the lack of gender differences in the mediation model suggests that once anxiety is experienced, the ensuing psychological processes of resource depletion and self-concept deterioration operate similarly for both boys and girls. This implies that the core intervention targets identified—bolstering psychological capital and self-concept—are likely to be universally beneficial for this population, irrespective of gender-specific anxiety baselines.

## Limitations and future directions

Several limitations should be acknowledged. First, the cross-sectional design precludes causal inferences. Longitudinal or experimental studies are needed to establish temporal precedence and strengthen causal claims. Second, reliance on self-report measures may introduce common method variance, although statistical tests indicated it was not a major concern. Future research could benefit from multi-method assessments, including teacher or parent reports. Third, the sample was drawn from a single city, which may limit generalizability. Replication with more diverse samples is warranted.

## Conclusion

This study provides a significant advance in understanding the psychological mechanisms of academic burnout among mid-primary school migrant children. Its core theoretical contribution is the articulation and empirical validation of a chain-mediation model, which delineates a specific sequence: migration-related anxiety depletes positive psychological capital, which in turn weakens self-concept, thereby leading to increased academic burnout. This model integrates strength-based and cognitive perspectives, moving beyond viewing protective factors as mere correlates to delineating their interdependent roles within a causal pathway. The findings offer a robust empirical foundation for designing targeted, multi-level interventions. To effectively mitigate academic burnout, support systems must extend beyond anxiety reduction to proactively and concurrently foster children's positive psychological resources (e.g., hope, resilience) and cultivate a stable, positive self-concept, thereby interrupting the identified detrimental cycle at multiple points.

## Data Availability

The datasets presented in this study can be found in online repositories. The names of the repository/repositories and accession number(s) can be found in the article/supplementary material.
